# Insulin resistance and hyperandrogenism drive steatosis and fibrosis risk in young females with PCOS

**DOI:** 10.1371/journal.pone.0186136

**Published:** 2017-11-21

**Authors:** Salvatore Petta, Alessandro Ciresi, Jessica Bianco, Vincenzo Geraci, Roberta Boemi, Luigi Galvano, Franco Magliozzo, Giovanni Merlino, Antonio Craxì, Carla Giordano

**Affiliations:** 1 Section of Gastroenterology, Di.Bi.M.I.S., University of Palermo, Palermo, Italy; 2 Section of Endocrinology, Diabetology and Metabolism, Di.Bi.M.I.S., University of Palermo, Palermo, Italy; 3 Medicina Generale, Palermo, Italy; John Hopkins University School of Medicine, UNITED STATES

## Abstract

**Background and aims:**

Nonalcoholic fatty liver disease (NAFLD) and polycystic ovary syndrome (PCOS) recognize obesity and insulin resistance (IR) as common pathogenic background. We assessed 1) whether PCOS is a risk factor for steatosis, and 2) the impact, in PCOS patients, of IR and hyperandrogenism on steatosis and fibrosis.

**Methods:**

We considered 202 consecutive Italian PCOS nondiabetic patients and 101 age-matched controls. PCOS was diagnosed applying the Rotterdam diagnostic criteria. Steatosis was diagnosed if hepatic steatosis index (HSI) >36, while fibrosis by using the FIB-4 score. As surrogate estimate of insulin sensitivity we considered the insulin sensitivity index (ISI). Free androgen index (FAI) was calculated as estimate of biochemical hyperandrogenism.

**Results:**

In the entire population, steatosis was observed in 68.8% of patients with PCOS, compared to 33.3 of controls (p<0.001), this association being maintained after adjusting for metabolic confounders (OR 3.73, 95% CI 1.74–8.02; P = 0.001). In PCOS patients, steatosis was independently linked to WC (OR 1.04, 95% CI 1.01–1.08; P = 0.006) and ISI Matsuda (OR 0.69, 95% CI 0.53–0.88; P = 0.004), not to free androgen index (OR 1.10, 95% CI 0.96–1.26; P = 0.14). Notably, ISI Matsuda was confirmed as independently associated with steatosis in both obese (OR 0.42, 95% CI 0.23–0.77, P = 0.005) and nonobese (OR 0.69, 95% CI 0.53–0.91, P = 0.009), patients, while FAI (OR 1.45, 95% CI 1.12–1.87; P = 0.004) emerged as an independent risk factor only in nonobese PCOS. Similarly, higher FIB-4 was independently associated with higher FAI (p = 0.02) in nonobese and with lower ISI Matsuda (p = 0.04) in obese patients.

**Conclusions:**

We found that PCOS is an independent risk factor for steatosis, and that, IR and hyperandrogenism, this last especially in nonobese patients, are the key players of liver damage in PCOS.

## Introduction

Non-alcoholic fatty liver disease (NAFLD) is diagnosed in roughly 20–30% of the general population worldwide [1], its epidemic being related to the emerging increase in obesity and diabetes. Consistently, NAFLD is becoming the most frequent etiology of chronic liver diseases [2], the emerging cause of hepatocellular carcinoma [3], the leading indication for liver transplantation [4], and a risk factor for cardiovascular complications [5].

Male gender, obesity, insulin resistance (IR) and diabetes [6] are the most relevant risk factors associated with presence of NAFLD and with its severity in terms of steatohepatitis (NASH) and severity of fibrosis, this last being the main predictor of hepatic and extrahepatic prognosis in NAFLD patients [7]. Regarding gender, males are considered at higher risk of NAFLD and its severity when compared to fertile women [8], while menopausal women have a risk similar to that observed in men, due to the lack of the metabolic and hepatic protective effect of estrogens [9–10].

Available evidence however suggests that among fertile women, those affected by polycystic ovary syndrome (PCOS) could be at increased risk of liver damage. Observational studies showed a higher prevalence of fatty liver diagnosed by ultrasound (US), noninvasive scores or magnetic resonance spectroscopy (MRI) in PCOS patients compared with controls [11–13]. Besides, in PCOS women, insulin resistance (IR) and hyperandrogenism have been identified as the principal risk factors for steatosis and liver damage, assessed by noninvasive scores, even if these associations were not confirmed after adjusting for metabolic and hepatic risk factors [12–17].

In this view, in a large cohort of PCOS patients and of age-matched controls, we assessed whether PCOS firstly represents a risk factor for steatosis, and secondly whether insulin resistance and hyperandrogenism exerts a specific role in determining steatosis and fibrosis in affected women.

## Patients and methods

### Patients

The study included 202 consecutive White women, aged 20–51 years (mean 33 ± 5) followed up in PCOS Outpatients Clinic of the Section of Endocrinology, Diabetology and Metabolic Diseases (from January 1st 2005 to December 31st 2015). PCOS was defined by the modified Rotterdam criteria, after excluding other endocrine disorders. All PCOS women had the presence of at least two criteria among clinical (hirsutism and/or other signs and symptoms of hyperandrogenism, i.e. acne/seborrhea and alopecia) and/or biochemical hyperandrogenism, ovulatory dysfunction and polycystic ovarian morphology [18].

The following subjects were excluded from the study: women treated with clomiphene citrate, oral contraceptives, antiandrogens, drugs to control their appetite or insulin-sensitizing drugs during the 6 months before the examination; women with hyperprolactinemia; patients with basal 17-OH progesterone levels >6.05 nmol/l and peak >30.26 nmol/l at 60 min after 250 mg Synacthen; women with DHEAS >16.32 mmol/l who presented adrenal hyperplasia or adenoma or virilizing androgen-secreting neoplasias; and women whose clinical and hormone evaluation suggested Cushing's syndrome.

We also excluded from the analysis women with evidence of viral infection (anti-HCV, anti-HIV, and HBsAg negativity) and a previous history of excessive alcohol consumption.

Control fertile women, age-matched 1:2 to PCOS patients, were referred from general practitioners. They were part of an ongoing project aimed at assessing cardiovascular risk and liver damage in the general population, according to presence or absence of fatty liver.

All women enrolled as controls had available an abdominal and pelvic ultrasound performed during the last year, aimed at seeing both liver and ovarian morphology.

They had no previous history of symptomatic cardiovascular disease (transient ischemic attack, stroke, angina, myocardial infarction, right or left ventricular dysfunction), no evidence of viral infection (anti-HCV, anti-HIV, and HBsAg negativity), alcohol consumption <20 g/day during the previous year (evaluated by a specific questionnaire). The diagnosis of PCOS, in accordance with Rotterdam criteria [18], was excluded in all control subjects. Particularly, control women had regular menstrual cycle, no clinical signs of hyperandrogenism and no polycystic ovarian morphology.

The study was carried out in accordance with the principles of the Helsinki Declaration, and with local and national laws. Approval was obtained from the AOUP “Giaccone” of Palermo Internal Review Board and its Ethic Committee, and written informed consent for the study was obtained from all controls and patients.

### Clinical and laboratory assessment

Clinical and anthropometric data were collected at the time of the enrollment. Body mass index (BMI) was calculated on the basis of weight in kilograms and height in meters according to the formula Kg/m^2^.

Obesity was defined as BMI ≥30 Kg/m^2^. Waist circumference (WC) was measured at the midpoint between the lower border of the rib cage and the iliac crest.

Abdominal obesity was defined according to ATPIII criteria (WC > 88 cm). A 12-hour overnight fasting blood sample was drawn at the time of enrollment to determine serum levels of AST/ALT, PLT, total cholesterol, HDL-cholesterol, triglycerides.

Patients were tested for FSH, LH, 17-β-E2, 17-OH-Pg, basal PRL, total and free testosterone, DHEA sulphate (DHEAS), Δ4androstenedione, total cholesterol, high-density lipoprotein (HDL) cholesterol, low-density lipoprotein (LDL) cholesterol and triglycerides during the follicular phase (day 7 from the beginning of the last period). Progesterone levels were assessed during the luteal phase (day 21 from the beginning of the last period).

The following hormonal parameters were evaluated for the diagnosis of biochemical hyperandrogenism: total testosterone, DHEAS, Δ4-androstenedione and sex hormone-binding globulin (SHBG) (data not shown). FAI was calculated as the ratio of total testosterone levels in nmol/l to SHBG levels in nmol/lt×100 (%) [19].

Transvaginal ovarian ultrasound scanning were performed on day 10 from the beginning of the last period using a 7·5- MHz vaginal probe transducer (General Electric LOGIQ 400MD, Milwaukee, WI). Both ovaries were measured in the sagittal, transverse and coronal plane. Ovaries were classified as polycystic based on the presence of 12 or more follicles in each ovary measuring 2–8 mm in diameter, and/or increased ovarian volume (> 10 ml) [18].

Serum glucose and insulin levels at 0, 30, 60, 90, and 120 min during oral glucose tolerance test (OGTT, 75 g glucose) were assessed in all PCOS patients on the same day as other metabolic evaluations. As surrogate estimates of insulin sensitivity we calculated the insulin sensitivity index (ISI), a composite index derived from the OGTT and validated by Matsuda and DeFronzo [20].

#### Noninvasive assessment of steatosis and fibrosis

In both PCOS patients and controls, steatosis was diagnosed by using the validated hepatic steatosis index (HSI) that takes into account the following parameters: BMI, AST/ALT, gender, diabetes [21]. The score was calculated using the original reported formula, and patients where considered with steatosis if HSI > 36 [21].

In PCOS patients, fibrosis was assessed by using the validated FIB-4 score that takes into account the following parameters: age, AST, ALT, PLT [21]. The score was calculated using the original reported formula, and patients where considered with severe fibrosis if FIB-4 > 2.67 [22].

#### Assays

All hormones were measured in our laboratory using commercial kits. Serum insulin was assessed by electrochemiluminescence immunoassay (ECLIA) (Roche Diagnostic), with a lower detection limit of 0.2 μUI/ml (< 1.39 pmol/L). Serum total testosterone was assessed by ECLIA (Roche Diagnostic), with a lower detection limit of < 2.50 ng/dL (< 0.087 nmol/L). Δ4-androstenedione was assessed by ELISA (ng/ml; Arnika, Milan, Italy; analytical sensitivity: 0.021 ng/ml). Chemiluminescence assays were used for DHEAS (μg/dl; Immulite, Diagnostic Products, Genoa, Italy; analytical sensitivity: 15 μg/dl) and serum SHBG (nmol/l; Immulite, Diagnostic Products, Genoa, Italy; analytical sensitivity: 0.015 nmol/l). Serum glucose levels were measured using an electrochemical system (Glucocard, Menarini Diagnostics, Florence, Italy). Total cholesterol, HDL, triglycerides, AST and ALT were measured in our laboratory using standard assays. LDL cholesterol levels were calculated with Friedewald's formula.

### Statistics

Continuous variables were summarized as mean ± standard deviation, and categorical variables as frequency and percentage. The t-test and chi-square test were used when appropriate. Univariate and multiple logistic regression models were used to assess the factors independently associated with presence of steatosis (HSI > 36) in the entire cohort of PCOS and controls, and in the group of PCOS patients. Univariate and multiple linear regression model was used to assess the factors independently associated with fibrosis by FIB-4 as continuous variable in PCOS patients.

As candidate risk factors, we selected age (only for steatosis), waist circumference, total cholesterol, HDL cholesterol, triglycerides, blood glucose, PCOS (only for steatosis in the entire population), insulin (only in analyses in PCOS), ISI Matsuda (only in analyses in PCOS), and Free androgen index (only in analyses in PCOS). Variable significant at univariate analysis (p<0.10) were included in multivariate models. P<0.05 was considered to be statistically significant.

Regression analyses were performed using SPSS version 19.

## Results

### Patient features

The baseline features of the 202 PCOS patients and of 101 age-matched controls are shown in Table 1.

**Table 1:** Clinical and metabolic characteristics of patients with PCOS and controls.

VARIABLE	PCOS (n = 202)	CONTROLS (n = 101)	*p*
	*Mean ± SD*	*Mean ± SD*	
**Age (years)**	33.2 ± 5.5	34.9 ± 8.2	0.070
**BMI (Kg/m2)**	25.7 ± 2.9	23.9 ± 3.0	0.001
**WC (cm)**	87.5 ± 22.0	81.7 ± 14.6	0.007
**Fasting glucose (mg/dl)**	88.9 ± 23.0	89.9 ± 15.3	0.654
**Total cholesterol (mg/dl)**	181.4 ± 37.0	184.8 ± 39.0	0.528
**HDL cholesterol (mg/dl)**	50.9 ± 13.1	62.6 ± 17.1	0.001
**Triglycerides (mg/dl)**	112.6 ± 44.0	98.2 ± 42.8	0.001
**AST (U/l)**	20.4 ± 7.3	17.0 ± 4.1	0.001
**ALT (U/l)**	21.7 ± 12.3	15.8 ± 4.4	0.001

Abbreviations: BMI, body mass index; WC, waist circumference; HDL, high density lipoprotein; AST, aspartate aminotransferase; ALT, alanine aminotransferase; PCOS, polycystic ovary syndrome.

PCOS patients had higher BMI (25.7±2.9 vs 23.9±3.0 Kg/m^2^, p<0.001) and a higher prevalence of abdominal obesity (48.2% vs 34.6%, p = 0.01) when compared to controls, as well as lower HDL (50.9±13.1 vs 62.6±17.1 mg/dl, p<0.001) and higher triglycerides levels (112.6±44.0 vs 98.2±42.8 mg/dl, p = 0.01), and higher AST (20.4±7.3 vs 17.0±4.1 U/l, p<0.001) and ALT (21.7±12.3 vs 15.8±4.4 U/l, p<0.001) values.

### PCOS is a risk factor for steatosis

In the entire population of PCOS and controls, steatosis, defined as HSI>36, was observed in 59% of cases (173/303).

The presence of steatosis was significantly associated with higher BMI, higher WC, and lower HDL and triglycerides levels (p<0.01 for all) (Table 2). Notably, steatosis was observed in 68.8% (139/202) of patients with PCOS, compared to 33.3 (34/101) of controls (p<0.001) (Table 2).

**Table 2:** Univariate and multivariate logistic regression analyses of factors associated with steatosis (HSI>36) in the entire population of PCOS and controls.

	HSI ≤ 36 (n = 130)	HSI > 36 (n = 173)	*p*	OR 95% C.I. *p*
	*Mean ± SD*	*Mean ± SD*		
**Age** (years)	34.4 ± 7.0	33.31 ± 6.23	0.160	-
**BMI** (Kg/m2)	23.4 ± 2.4	26.52 ± 2.82	<0.001	-
**WC** (cm)	79.4 ± 16.6	90.26 ± 21.12	<0.001	1.03 1.01–1.05 0.001
**Abdominal Obesity** [n (%)]	[37 (28.4)]	[93 (53.7)]	<0.001	
**Fasting glucose** (mg/dL)	91.4 ± 30.5	87.66 ± 10.73	0.210	-
**Total cholesterol** (mg/dl)	182.0 ± 41.9	182.64 ± 34.36	0.900	-
**HDL cholesterol** (mg/dl)	57.2 ± 15.0	50.90 ± 14.23	0.001	1.00 0.98–1.03 0.66
**Triglycerides** (mg/dl)	88.6 ± 41.6	122.0 ± 40.6	<0.001	1.01 1.00–1.02 0.001
**AST** (U/l)	18.0 ± 5.8	20.13 ± 6.97	0.010	-
**ALT** (U/l)	19.1 ± 9.6	19.56 ± 11.00	0.740	-
**PCOS** [n (%)]	[63 (48.4)]	139 (80.3)]	<0.001	3.73 1.74–8.02 0.001

Abbreviations: HIS, hepatic steatosis index; BMI, body mass index; WC, waist circumference; HDL, high density lipoprotein; AST, aspartate aminotransferase; ALT, alanine aminotransferase; PCOS, polycystic ovary syndrome.

**Fig 1:**
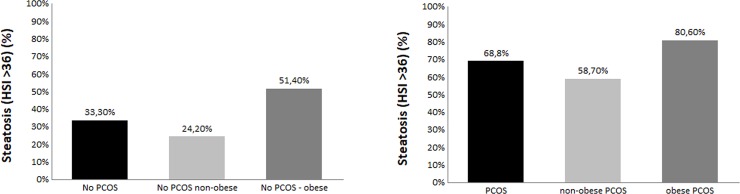
Prevalence of steatosis—defined as hepatic steatosis index > 36—according to presence of polycystic ovary syndrome (PCOS) and/or abdominal obesity.

Fig 1 shows the prevalence of steatosis according to the presence of PCOS and abdominal obesity.

By multivariate logistic regression analysis higher WC (OR 1.03, 95% CI 1.01–1.05; P = 0.001), higher triglycerides (OR 1.01, 95% CI 1.00–1.02; P = 0.001), and presence of PCOS (OR 3.73, 95% CI 1.74–8.02; P = 0.001) were risk factors independently associated with presence of fatty liver (Table 2).

### Determinants of steatosis and fibrosis in PCOS patients

Among patients with PCOS, those with steatosis, defined as HSI>36, showed significantly higher BMI (26.5±2.8 vs 24.1±2.4 Kg/m^2^, p<0.001), WC (93.2±17.3 vs 80.2±11.9 cm, p<0.001), fasting insulin (17.1±13.1 vs 9.6±3.4, p<0.001) and FAI (5.52±4 vs 3.18±2.7, p<0.001) and lower HDL cholesterol (49.7±12.8 vs 54.7±11.2 mg/dl, p = 0.011) and SHBG levels (50.4±23 vs 82.9±41.9 nmol/l, p<0.001) than patients without steatosis, without significant difference in fasting glucose (87.7±11 vs 91.5±17.7 mg/dl, p = 0.441), LDL cholesterol (110.1±29.7 vs 106.4±43 mg/dl, p = 0.484), triglycerides (102.9±54.4 vs 88.7±50.7 mg/dl, p = 0.083), DHEAS (7.8±3.7 vs 8.2±5.2 μg/dl, p = 0.539) and Δ4-androstenedione (10.4±7.5 vs 9.8±7.2 ng/ml, p = 0.668). Steatosis was significantly associated with higher BMI, higher WC, lower HDL lower ISI Matsuda index and higher FAI (p<0.10 for all), even if only WC (OR 1.04, 95% CI 1.01–1.08; P = 0.006) and ISI Matsuda (OR 0.68, 95% CI 0.53–0.88; P = 0.004) were confirmed at multivariate analysis, not FAI (OR 1.10, 95% CI 0.96–1.26; P = 0.14) (Table 3).

**Table 3:** Multivariate logistic regression analyses of factors associated with steatosis (HSI>36) in the entire population of PCOS, and in sub-groups according to presence/absence of abdominal obesity.

VARIABLE	OR	95% C.I.	*p*
	**PCOS (n = 202)**		
**Age** (years)	0.97	0.90–1.05	0.490
**WC** (cm)	1.04	1.01–1.08	0.006
**HDL cholesterol** (mg/dl)	0.99	0.96–1.03	0.810
**ISI Matsuda**	0.68	0.53–0.88	0.004
**FAI**	1.10	0.96–1.26	0.140
	**NONOBESE PCOS (n = 63)**		
**HDL cholesterol** (mg/d)	0.96	0.92–1.01	0.130
**ISI Matsuda**	0.69	0.53–0.91	0.009
**FAI**	1.45	1.12–1.87	0.004
	**OBESE PCOS (n = 139)**		
**Age** (years)	0.99	0.88–1.12	0.950
**ISI Matsuda**	0.42	0.23–0.77	0.005

Abbreviations: WC, waist circumference; HDL, high density lipoprotein; ISI, insulin sensitivity index; FAI, free androgen index; PCOS, polycystic ovary syndrome.

Notably, when splitting the population according to the presence/absence of abdominal obesity, steatosis was present in 75/93 (80.6%) obese and 64/109 (58.7%) non obese patients (p<0.001) (Fig 1) and while ISI Matsuda was confirmed as independently associated with steatosis in both groups (OR 0.69, 95% CI 0.53–0.91, P = 0.009 in non obese, OR 0.42, 95% CI 0.23–0.77, P = 0.005 in obese), higher FAI (OR 1.45, 95% CI 1.12–1.87; P = 0.004) emerged as an independent risk factor only in nonobese PCOS (Table 3).

None PCOS patients had a FIB-4 >2.67 suggestive of severe hepatic fibrosis. Consistently, we assessed variables associated with higher FIB-4 considered as continuous variable. FIB-4 was significantly associated with higher WC, lower HDL lower ISI Matsuda index and higher FAI (p<0.10 for all), even if only higher FAI (P = 0.04) (Table 4 up) was confirmed at multivariate analysis. Notably, when splitting the population according to the presence/absence of abdominal obesity, higher FIB-4 was independently associated with FAI in non obese (p = 0.02), while with ISI Matsuda (p = 0.04) in obese patients (Table 4).

**Table 4:** Multivariate linear regression analyses of factors associated with FIB-4 in the entire population of PCOS, and in sub-groups according to presence/absence of abdominal obesity.

VARIABLE	Beta	Standard error	*p*
		**PCOS (n = 202)**	
**WC** (cm)	0.144	0.002	0.29
**HDL Cholesterol** (mg/dl)	-0.065	0.003	0.60
**ISI Matsuda**	-0.121	0.026	0.42
**FAI**	0.246	0.013	0.04
		**NONOBESE PCOS (n = 63)**	
**FAI**	0.370	0.013	0.02
		**OBESE PCOS (n = 139)**	
**Triglycerides** (mg/dl)	-0.039	0.001	0.80
**ISI Matsuda**	-0.355	-0.052	0.04
**FAI**	0.164	0.017	0.35

Abbreviations: WC, waist circumference; HDL, high density lipoprotein; ISI, insulin sensitivity index; FAI, free androgen index; PCOS, polycystic ovary syndrome.

## Discussion

In the present study, performed in a large cohort of PCOS women and age-matched controls, we found that PCOS doubles the risk for steatosis and that, in PCOS patients, IR and hyperandrogenism are the two main determinants of steatosis and liver damage.

Available data [11–13] and a recent meta-analysis [23] reported that the prevalence of fatty liver, assessed by US, noninvasive scores, or MRI, is higher in patients with PCOS compared to controls. However, only a small study on about 60 patients corrected this association for metabolic confounders [24]. In the present study, we observed that in patients with PCOS, after adjusting for metabolic risk factors, the risk for steatosis -evaluated by the HIS- was three times higher compared to age-matched controls, these results being similar to those recently reported by Ayonrinde and colleagues [25].

In addition, it is interesting to note that our patients with PCOS and steatosis showed lower SHBG levels and this finding is in agreement with the well-known data on the relationship between the liver production of SHBG and metabolic parameters [26].

A bias of this result could be represented by the higher BMI and WC in women with PCOS than controls. However, since overweight or overt obesity are common features in patients with PCOS, we decided to consecutively include all PCOS women in order to avoid the bias to exclude those with higher BMI. In addition, notably, this result was maintained after adjusting for metabolic risk factors including lipid status and abdominal obesity, identifying PCOS as an independent risk factor for steatosis.

The above quoted data clearly highlight that PCOS patients, even if fertile and females, are at high risk of steatosis, so the identification of risk factor for fatty liver and for hepatic damage in this population is demanded. Different lines of evidences reported that classical risk factors for steatosis like obesity and IR are the main trigger of steatosis also in PCOS patients [12, 14–16, 27].

Moreover, other studies identified hyperandrogenism as an additional risk factor for fatty liver in PCOS [16]. Along this line, in NAFLD-related PCOS, obesity, IR and hyperandrogenism have been associated with NASH and fibrosis evaluated by noninvasive scores [13,17]. However, these studies did not stratify the population according to obesity, and in addition reported simply associations without adjusting for all confounders. In the present study, we confirmed IR and hyperandrogenism as two key players for steatosis and fibrosis in NAFLD patients with PCOS. However, when stratifying patients according to abdominal obesity, hyperandrogenism emerged as a key risk factor for both steatosis and fibrosis especially in nonobese at lower metabolic risk patients where the weight of IR is less pronounced.

Though this study was not designed to clarify the causal relationship between PCOS, steatosis and fibrosis, some hypotheses can be put forward in view of literature evidence. NAFLD and PCOS recognize the same dysmetabolic pathogenic background, i.e. obesity and IR [28, 29].In this scenario, PCOS-related hyperandrogenism may contribute to liver disease by promoting systemic inflammation leading to impairment in insulin sensitivity and liver fibrogenesis [28]. On the other hand, NAFLD could further implement this vicious circle by contributing to IR, a key element in PCOS pathogenesis [28].

From a clinical point of view, our study suggests that PCOS patients should be assessed for presence of steatosis, especially those with reduced insulin sensitivity and/or hyperandrogenism. In our study, no patients had a FIB-4 suggestive for severe fibrosis, this issue being probably due to the fact that our population is young and without diabetes. However, it is plausible that the persistence of IR and hyperandrogenism over time could prompt fibrosis progression, and consistently these patients should be careful followed looking at the correction of the above quoted risk factors.

The main limitation of this study lies in its cross-sectional nature, making it impossible to dissect the temporal relation between PCOS, IR, hyperandrogenism and steatosis/liver damage. A further methodological question is the use of noninvasive markers for detection of fatty liver and staging fibrosis, instead of liver biopsy. However, both HSI [30] and FIB-4 [31] have been largely validated for the noninvasive assessment of NAFLD, while liver biopsy is invasive with some time life-threating complications and cannot widely proposed in young PCOS patients and healthy controls. Finally, our study included a cohort of Italian PCOS patients, followed at a tertiary referral center, who may be different, in terms of both metabolic features and severity of liver disease, from the majority of PCOS cases n the general population.

In conclusion this study, performed on a large cohort of PCOS patients and age-matched controls, showed that PCOS is an independent risk factor for steatosis, and that IR and hyperandrogenism, this last especially in nonobese patients, are the key players of liver damage in PCOS.

The authors wish to dedicate this manuscript to the memory of Dr. Marco Calogero Amato, unforgettable researcher who dedicated his short career to the study of metabolism in PCOS and who taught his students to listen to the woman suffering from PCOS not only for what she appears but also for what she feels.

## Abbreviations

PCOS: polycystic ovary syndrome
ISI: insulin sensitivity index
FAI: free androgen index
IR: insulin resistance
NAFLD: non-alcoholic fatty liver disease
